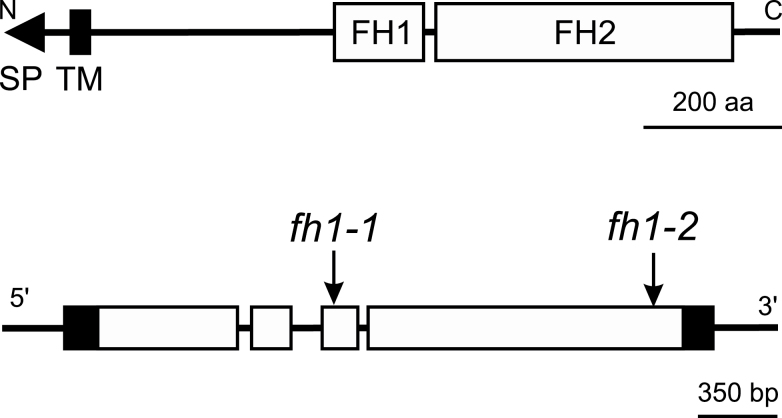# AtFH1 formin mutation affects actin filament and microtubule dynamics in *Arabidopsis thaliana*

**DOI:** 10.1093/jxb/erv473

**Published:** 2015-10-22

**Authors:** Amparo Rosero, Viktor Žárský, Fatima Cvrčková

**Affiliations:** 1 Department of Experimental Plant Biology, Faculty of Sciences, Charles University, Czech Republic; 2 Institute of Experimental Botany, Academy of Sciences of the Czech Republic, Czech Republic

Journal of Experimental Botany, Vol. 64, No. 2, pp. 585–597, 2013, doi: 10.1093/jxb/ers351.

While preparing a follow-up to the above article, we have realised that a wrong position of one of the T-DNA insertions within the AtFH1 (At3g25500) locus has been provided in Figure 1A and subsequently also in Results in the above paper. The corrected Figure 1A is provided here. The *fh1-2* insertion is located at the 3′ end of the ORF, allowing for production of a FH1 protein C-terminally truncated by about 50 amino acids but preserving an intact FH2 domain, and thus likely to be at least partly functional. This error does not affect any other data in the paper (including the description of the mutant lines in Materials and methods) or any of its conclusions (including the interpretation of the *fh1-2* allele as a partial loss of function mutation). The authors apologize for any inconveniences this error might have caused.

**Figure:**